# European Drug Users at Risk from Illicit Fentanyls Mix

**DOI:** 10.3389/fphar.2017.00785

**Published:** 2017-10-31

**Authors:** Simona Pichini, Roberta Pacifici, Enrico Marinelli, Francesco P. Busardò

**Affiliations:** ^1^National Centre on Addiction and Doping, Istituto Superiore di Sanità, Rome, Italy; ^2^Unit of Forensic Toxicology, Department of Anatomical, Histological, Forensic and Orthopedic Sciences, Sapienza University, Rome, Italy

**Keywords:** fentanyl derivatives, intoxications, fatalities, new synthetic opioids, health threat

The increase of overdose deaths involving licit and illicit fentanyl analogs (FAs), recently observed in North America, has shifted toward Europe posing a serious public health menace (Mounteney et al., 2015).

The widespread appearance of these synthetic opioids, between 50 and 100 times more potent than morphine and in many cases not approved for medical use, has been reported by the European Monitoring Centre for Drugs and Drug Addiction (European Drug Report, 2017)^1^.

Indeed, in the last few years a number of FAs, (e.g., despropionyl-2-fluorofentanyl, furanylfentanyl, valerylfentanyl, acryloylfentanyl, carfentanyl, butyrfentanyls) has appeared for the first time on the European illicit market having caused more than 100 fatalities, when used alone or in association to other drugs (UNODC, 2017).

Due to the low cost of the required materials and equipment for producing these compounds in clandestine laboratories inside and outside Europe, they are sold by drug dealers in place of heroin or mixed with it as cutting agents. In this context, the possibility of fatal overdoses is extremely high because of the narrow range between a safe and a lethal dose and the manufacturing of quantitatively inaccurate and contaminated products (UNODC, 2017).

Current evidence suggests that both FAs availability on the illicit market and related acute and lethal intoxications are indisputably underestimated because of the analytical challenges caused by the structural difficulties in identifying unscheduled compounds. Often, overdoses caused by novel synthetic opioids are not fully investigated, difficult to report according to ICD 10 (especially in the way to distinguish different fentanyl analogs) and are simply classified as “heroin-related fatalities” (Swanson et al., 2017). Additional challenge could be also that the range of FAs being used is continuously changing and for health professionals, forensic toxicologists, pathologists, epidemiologists and lawmakers it is difficult to be up to date. Recently, Frank and Pollack commented on the threat to public health caused by widespread use of illegal fentanyl in US with the doubling of overdose deaths involving this drug (Frank and Pollack, 2017). The authors suggested to primarily implement policies of “harm reduction” redirecting user demand away from products containing fentanyl, reducing unintentional fentanyl consumption, increasing penalties on illegal fentanyl distributors and sellers and finally providing timely availability of naloxone and medication-assisted therapy. In this concern, there is a suggestion by health professionals to increase naloxone doses in case of overdoses by fentanyl analogs.

In addition to these recommendations, we wish to draw the attention of the whole scientific community on the importance of improving surveillance and data sharing of FAs through National and International Early Warning Systems (EWS) set up throughout Europe and a standardization in reporting FAs related deaths. Analytical methodologies for the identification of fentanyl analogs and eventual metabolites in ante mortem and post mortem cases should be developed, validated, and analytical data shared through different communication platforms (e.g., EWS and Europol).

We advocate for the correct identification of the phenomenon to implement contextual strategies in order to restrain the use and abuse of FAs and stop this incoming threat for health of European drug users.

## Author contributions

FPB prepared the first daft of this opinion paper together with the other three coauthors, revising the existing literature and approved the last draft of the paper. SP, RP, and EM revised the existing literature concerning fentanyls, prepared the first draft of the opinion with FPB and approved the final version.

### Conflict of interest statement

The authors declare that the research was conducted in the absence of any commercial or financial relationships that could be construed as a potential conflict of interest.

## Footnotes

^1^http://www.emcdda.europa.eu/edr2017 (last accessed July 13, 2017).
